# Study protocol: The back pain outcomes using longitudinal data (BOLD) registry

**DOI:** 10.1186/1471-2474-13-64

**Published:** 2012-05-03

**Authors:** Jeffrey G Jarvik, Bryan A Comstock, Brian W Bresnahan, Srdjan S Nedeljkovic, David R Nerenz, Zoya Bauer, Andrew L Avins, Kathryn James, Judith A Turner, Patrick Heagerty, Larry Kessler, Janna L Friedly, Sean D Sullivan, Richard A Deyo

**Affiliations:** 1 Department of Radiology, University of Washington, Seattle, WA, USA; 2 Department of Neurological Surgery, University of Washington, Seattle, WA, USA; 3 Comparative Effectiveness, Cost and Outcomes Research Center, University of Washington, Seattle, WA, USA; 4 Department of Anesthesiology, Perioperative and Pain Medicine, Brigham and Women’s Hospital, Boston, MA, USA; 5 Neuroscience Institute, Henry Ford Hospital, Detroit, MI, USA; 6 Division of Research, Kaiser Permanente Northern California, Oakland, CA, USA; 7 Department of Health Services, University of Washington, Seattle, WA, USA; 8 Department of Pharmacy, University of Washington, Seattle, WA, USA; 9 Departments of Family Medicine and Internal Medicine, Oregon Health Sciences University, Portland, OR, USA

**Keywords:** Low back pain, Registry, Cohort studies, Aged, Primary health care

## Abstract

**Background:**

Back pain is one of the most important causes of functional limitation, disability, and utilization of health care resources for adults of all ages, but especially among older adults. Despite the high prevalence of back pain in this population, important questions remain unanswered regarding the comparative effectiveness of commonly used diagnostic tests and treatments in the elderly. The overall goal of the Back pain Outcomes using Longitudinal Data (BOLD) project is to establish a rich, sustainable registry to describe the natural history and evaluate prospectively the effectiveness, safety, and cost-effectiveness of interventions for patients 65 and older with back pain.

**Methods/design:**

BOLD is enrolling 5,000 patients ≥ 65 years old who present to a primary care physician with a new episode of back pain. We are recruiting study participants from three integrated health systems (Kaiser-Permanente Northern California, Henry Ford Health System in Detroit and Harvard Vanguard Medical Associates/ Harvard Pilgrim Health Care in Boston). Registry patients complete validated, standardized measures of pain, back pain-related disability, and health-related quality of life at enrollment and 3, 6 and 12 months later. We also have available for analysis the clinical and administrative data in the participating health systems’ electronic medical records. Using registry data, we will conduct an observational cohort study of early imaging compared to no early imaging among patients with new episodes of back pain. The aims are to: 1) identify predictors of early imaging and; 2) compare pain, functional outcomes, diagnostic testing and treatment utilization of patients who receive early imaging versus patients who do not receive early imaging. In terms of predictors, we will examine patient factors as well as physician factors.

**Discussion:**

By establishing the BOLD registry, we are creating a resource that contains patient-reported outcome measures as well as electronic medical record data for elderly patients with back pain. The richness of our data will allow better matching for comparative effectiveness studies than is currently possible with existing datasets. BOLD will enrich the existing knowledge base regarding back pain in the elderly to help clinicians and patients make informed, evidence-based decisions regarding their care.

## Background

Back pain is a particularly important problem for older adults. The prevalence of severe, disabling back pain increases in older adults [1,2]. Moreover, with an aging population, the importance of back pain in the U.S. will only increase in coming decades. Despite this, there is a paucity of research on back pain in older age, and most studies to date have been small [1].

The Back pain Outcomes using Longitudinal Data (BOLD) project establishes a large, community-based registry of patients aged 65 years and older presenting with new episodes of healthcare visits for back pain. BOLD’s primary aim is to establish an infrastructure that allows the conduct of prospective, controlled studies comparing the effectiveness of diagnostic and treatment strategies for back pain in the elderly. The importance of BOLD stems from the high prevalence, clinical impact and cost of back pain, combined with a relative lack of comparative effectiveness data, especially for older adults. Back pain, an Institute of Medicine priority condition [3], is one of the most important causes of functional limitations and disability among adults in the United States. Back pain is also one of the most common reasons for physician visits [4]. The economic impact of back pain is substantial. Martin et al., estimated that in 2005, the marginal direct cost of care for people with back pain compared to those without was over $86 billion [5].

Although there are numerous guidelines regarding the diagnosis and treatment of back pain in general, these evidence-based guidelines do not focus on the elderly. Age-related differences in the causes of back pain highlight the need for specific guidelines for diagnosing and treating back pain in older adults. For example, back pain due to metastatic cancer has a higher prevalence in older adults. In a study of primary care patients with back pain, age older than 50 years was associated with a higher likelihood of having cancer (positive likelihood ratio = 2.7), although the absolute probability of having cancer remained small at 1.2% [6]. This increased risk of cancer, as well the greater prevalence in older adults of other conditions such as spinal stenosis, vertebral compression fractures and aortic aneurysms, has led most guidelines to call for early diagnostic imaging in the elderly. However, it remains unclear how early imaging in the elderly affects clinical outcomes and costs associated with the treatment of back pain. A primary goal of the BOLD project is to enrich the existing knowledge base regarding back pain in the elderly to help clinicians and patients make informed, evidence-based decisions regarding their care.

## Methods/design

### BOLD registry

#### Overview

The overall goal of this project is to establish a sustainable and rich registry to evaluate prospectively the effectiveness, safety, and cost-effectiveness of diagnostic approaches and interventions for elderly patients with back pain. The registry can also be used to identify and recruit patients for additional studies. We plan to recruit 5,000 patients age 65 and older with new episodes of health care visits for back pain (defined as no prior visits to a health care provider for back pain care within 6 months). Patients who enroll in the registry complete validated, standardized measures of pain, back pain-related disability, and health-related quality of life at enrollment and 3, 6 and 12 months later. Our project includes a demonstration comparative effectiveness study of early (<six weeks after initial medical visit) imaging versus no early imaging for elderly patients with back pain. In this observational cohort study we will test the hypothesis that early imaging is associated with more interventions and adverse labeling (where simply assigning a diagnostic label results in worse health related quality of life), greater disability and higher levels of pain compared to matched controls who do not undergo early imaging. We will also test the hypothesis that racial and ethnic minorities will have lower rates of early imaging than non-minorities. In parallel with construction of the BOLD registry, we also are performing a double-blind, randomized controlled trial of epidural steroid with local anesthetic compared with a local anesthetic injection alone for spinal stenosis; this component of the study is described elsewhere [7].

#### Participating centers

BOLD is recruiting patients at three integrated health care systems: Kaiser Permanente of Northern California (KPNC), Henry Ford Health System (HFHS), and Harvard Vanguard Medical Associates/Harvard Pilgrim Health Care (HVMA/HPHC). We chose these sites for their geographic and demographic diversity. Confining our registry to the integrated health systems with comprehensive electronic medical record systems allows us to take advantage of the well-defined populations that their patients comprise as well as the wealth of data available in these systems, including health care utilization.

The University of Washington’s Comparative Effectiveness, Cost and Outcomes Research Center (CECORC) and Center for Biomedical Statistics (CBS) serve as the Data Coordinating Center (DCC) for BOLD. A collaborator at Oregon Health and Sciences University (RAD) is also part of the DCC.

#### Institutional review board (IRB) approval

The IRBs at all participating institutions (University of Washington, Harvard Vanguard, Harvard Pilgrim, Henry Ford Health System, and Kaiser-Permanente Northern California) reviewed and approved the protocols for the BOLD Registry and the Observational Cohort Study of Early Imaging.

#### Subject eligibility

We identify patients at their health care visits for back pain using the Ninth International Classification of Diseases (ICD-9) codes [8]. We recruit subjects from both primary care clinics and urgent care/emergency care settings. Since our aim is to evaluate treatment effectiveness (how an intervention performs in the real world) rather than efficacy (how an intervention performs under ideal conditions), our inclusion criteria are as broad as possible. Our inclusion and exclusion criteria are listed in Table 1.

**Table 1:** Inclusion and Exclusion Criteria

**Inclusion Criteria**	
1.	Age ≥ 65 years
2.	Primary care visit for back pain based on ICD9 code (Table 2)
**Exclusion criteria:**	
1.	Health care encounter for back pain within 6 months
2.	Previously contacted for registry participation
3.	Prior lumbar spine surgery
4.	Developmental spine deformities
5.	Inflammatory spondyloarthropathy
6.	Spinal malignancy or infection
7.	History of cancer within past 5 years excluding non-melanomatous skin cancer
8.	History of HIV within past 5 years
9.	No telephone
10.	Planning on leaving Health System within the next 12 months
11.	Unable to understand English
12.	Severe mental impairment that would interfere with answering questions

#### Patient identification

We screen for study eligibility patients ≥ 65 years old who have had a primary care visit (including by telephone) or urgent/emergency care visit and been assigned a diagnosis code indicating back pain within the past three weeks (Table 2). In addition to patient encounters with physicians, we also include patients who have had encounters with non-physician primary care providers (registered nurses, nurse practitioners and physician assistants). We select for patients with relatively new onset episodes of back pain by excluding those with visits for back pain in the previous 6 months.

**Table 2:** ICD9 Inclusion and Exclusion Codes for the Registry/Observational Cohort

**Inclusion Diagnoses**		**Exclusion Diagnoses**	
**ICD9 Code**	**Description**	**ICD9 Code**	**Description**
307.89	Psychogenic backache	V66.7	Encounter for palliative care
721.3	Lumbosacral spondylosis w/o myelopathy	042, 079.53	HIV within the last five years
		140-239.9 (except 173, 210–229)	Cancer other than non-melanoma skin cancer within the last 5 years and benign neoplasms
721.5	Kissing spine (Baastrup Disease)	290.0–290.4	Dementia
721.6	Ankylosing vertebral hyperostosis	324.1	Intraspinal abscess
721.7	Traumatic spondylopathy	331.0	Alzheimer’s disease
721.8	Other allied disorders of spine	630–676	Pregnancy-related diagnoses
721.9	Spondylosis of unspecified site w/o myelopathy	720.0–720.9	Inflammatory spondyloarthropathies
721.90	Spondylosis of unspecified site w/o myelopathy	730–730.99	Osteomyelitis
722.1	Displacement of thoracic or lumbar disc w/o myelopathy	737.30–737.39 737.40–737.43	Developmental spine deformities
722.10	Displacement of lumbar disc w/o myelopathy	733.8, 733.81–733.8InternalRef>)	Non-union/mal-union of fracture
722.11	Displacement of thoracic disc w/o myelopathy	805–806.9	Fractures of spinal column
722.2	Degeneration of intervertebral disc, site unspecified	839–839.59	All vertebral dislocations
722.3	Schmorl’s nodes	E800-E849.9	Vehicular accidents
722.31	Schmorl’s nodes- thoracic region	03.09; 80.50	Prior lumbar surgery:
722.32	Schmorl’s nodes- lumbar region	80.51; 80.52;	·Laminectomy
722.5	Degeneration of thoracic or lumbar intervertebral disc	80.59; 81.00;	·Discectomy
722.51	Degeneration of thoracic or thoracolumbar intervertebral disc	81.06–81.09;	·Fusion
722.52	Degeneration of lumbar or lumbosacral intervertebral disc	03.02; 03.6;	·Other
722.6	Degeneration of intervertebral disc, site unspecified	78.69	
722.9	Other and unspecified disc disorder		
722.90	Other and unspecified disc disorder of unspecified region		
722.92	Other and unspecified disc disorder of thoracic region		
722.93	Other and unspecified of lumbar region		
724	Other and unspecified disorders of back		
724.0	Spinal stenosis, not cervical		
724.00	Spinal stenosis of unspecified region		
724.01	Spinal stenosis- thoracic		
724.02	Spinal stenosis- lumbar		
724.03	Spinal stenosis- lumbar with neurogenic claudication		
724.09	Spinal stenosis- other region		
724.1	Pain in thoracic spine		
724.2	Lumbago		
724.3	Sciatica		
724.4	Back pain w/radiation, unspec		
724.5	Backache, unspecified		
724.6	Disorders of sacrum (including Lumbosacral junction)		
733.1	Pathologic fractures:		
733.10,	·unspecified site,		
733.13	·vertebrae		
733.95	Stress fracture of other bone		
738.4	Acquired spondylolisthesis		
738.5	Other acquired deformity of back or spine		
739.2	Nonallopathic lesions- thoracic, not elsewhere classified		
739.3	Nonallopathic lesions, lumbar, not elsewhere classified		
739.4	Nonallopathic lesions, sacral, not elsewhere classified		
756.11	Spondylolysis		
756.12	Spondylolisthesis		
846.0	Lumbosacral sprain		
846.1	Sacroiliac (ligament) sprain		
846.2	Sacrospinatus (ligament) sprain		
846.3	Sacrotuberous (ligament) sprain		
846.8	Other specified sites of sacroiliac region sprain		
846.9	Unspecified site of sacroiliac region sprain		
847.1	Thoracic sprain		
847.2	Lumbar sprain		
847.3	Sacral sprain		
847.9	Sprain- unspecified site of back		

#### Patient enrollment

The exact method of the initial subject contact varies slightly at each site. The site research staff identify and contact potential subjects by telephone, email, mail or in person, describing the study and inviting them to participate using a standardized script. In the invitation we provide a web address (http://www.backpainproject.org) that has additional information about the study.

If the patient agrees, the research staff determines eligibility, verifying inclusion/exclusion criteria that were assessed during the query of the electronic health information system (Table 3). Patients provide verbal assent for their participation in the registry, which includes access to their medical records.

**Table 3:** Additional Exclusion Criteria Assessed at Initial Telephone Contact

1.	Previously contacted for registry participation
2.	Severe cognitive impairment that would interfere with answering questions
3.	Tumor/cancer related to the spine
4.	Any history of cancer within past 5 years, other than non-melanomatous skin cancer
5.	Spine abscess
6.	Ankylosing spondylitis
7.	Spine deformity from childhood
8.	Spine fracture
9.	Vehicular accident as cause of back pain
10.	Prior lumbar spine surgery
11.	No telephone, or planning to leave health system within next 12 months
12.	Unable to understand English

We offer subjects a $10 gift card or check for each completed interview (baseline and 3, 6, and 12 months later). The total time for completing the study questionnaires at each assessment is approximately 15–30 minutes.

#### Data collection

Our data come primarily from two sources: subject questionnaires and electronic data records. We have attempted to minimize the questionnaire burden while still obtaining important information regarding the patient’s back pain. At baseline, trained research coordinators/interviewers administer the questionnaires either in person or over the telephone within three weeks of a subject’s index primary care visit.

#### Follow-up

We contact each registry patient at three, six and 12 months after baseline to collect data on patient treatments and outcomes. For follow-ups, the questionnaires are either self-administered by the subject using a mailed hard copy or administered by a research coordinator over the telephone. We plan to develop an on-line version of the questionnaire as an option for subjects to complete after being sent a link by email.

Follow-up questionnaires can be completed within a two-week window on either side of the follow-up time-point. We use a computerized tracking system to identify when patients enter the interview window and when interviews are complete. If patients withdraw from the study, we attempt to identify the reason.

#### Baseline and follow-up measures

We collect demographic information and information regarding back pain duration and back pain recovery expectations at baseline. We also administer the following measures at each assessment: 1) Roland-Morris Disability Questionnaire (RMDQ) [9], modified slightly to indicate disability due to back or leg pain (sciatica); 2) 0–10 numerical rating scales (NRS) of average back and leg pain in past 7 days; 3) Brief Pain Inventory activity interference scale; [10,11] 4) Patient Health Questionnaire (PHQ)-4 Depression and Anxiety screen; [12] 5) the EuroQol-5D (EQ5D) [13] 6) Behavioral Risk Factor Surveillance System (BRFSS) survey (2 questions about falls) [14]. We repeat the same measures at each follow-up time-point except for the duration of pain and patient recovery expectation questions.

#### Baseline descriptive measures

*Pain Duration:* We ask subjects at baseline to categorize the length of the current episode of back or leg pain (sciatica) as follows: 1) less than 1 month; 2) 1–3 months, 3) 3–6 months; 4) 6–12 months; 5) 1–5 years; and 6) more than 5 years.

*Patient Expectations:* We ask subjects to use a 0–10 NRS to rate their confidence that their pain will be completely gone or much better in 3 months.

#### Primary outcome measure

*Roland-Morris Disability Questionnaire:* Our primary outcome measure is the Roland-Morris Disability Questionnaire (RMDQ) [9], a back pain-specific functional status questionnaire adapted from the generic Sickness Impact Profile (SIP) [15]. The original version consists of 24 yes/no items, which represent common dysfunctions in daily activities experienced by patients with back pain [9] .We use a slightly modified version of the questionnaire in which we add “or leg (sciatica)” to the words “back pain” where appropriate. A single score is derived by summing the items endorsed by the respondent, with higher scores indicating worse function. Both the original and modified RMDQ have proven to be more responsive to change over time than most subscales of the SF-36 [16] or disability day questions from national health surveys [16]. Its internal consistency is excellent [17]. Its construct validity is supported by significant associations in the expected directions with symptom severity, neurologic deficits, opioid medication use, work absenteeism, and other measures of health status (subscales of the SF-36, disability days) [18,19]. The RMDQ was the measure most responsive to clinical changes over time in the Maine Low Back Pain Cohort study [16].

#### Additional patient-reported measures

*Pain Numerical Rating Scale (NRS):* We ask subjects to rate separately their average back and leg pain within the past seven days on 0–10 scales, with 0 = no pain and 10 = worst pain imaginable. Investigators commonly use NRS’s of pain intensity as outcomes in clinical trials of pain therapies, and these ratings have been demonstrated to be valid, reliable, and sensitive to detecting change in pain intensity after treatment [20]. The Initiative on Methods, Measurement, and Pain Assessment in Clinical Trials (IMMPACT) recommended a 0–10 NRS measure of pain intensity as a core outcome measure in pain clinical trials and noted that NRS measures had advantages over visual analogue scale (VAS) measures, including ability to be administered by telephone, preference by patients, and less missing and incomplete data [21]. Further, older adults may have difficulty completing VAS measures [20]. The IMMPACT group also recommended that clinical trials report the percentage of patients obtaining reductions in pain intensity from baseline of at least 30% on the NRS, and suggested that investigators may also wish to report the percentages of patients obtaining reductions in pain intensity of at least 50%. We plan to use both of these indicators of clinically meaningful change.

##### Pain Interference

The validated Brief Pain Inventory (BPI) Interference scale measures pain interference with activities [11].The scale consists of 7 ratings (0–10) of how much back pain interferes with the following: general activity, mood, ability to walk, normal work, relations with other people, sleep and enjoyment of life.

##### Patient Health Questionnaire-4 Depression and Anxiety Screen

The PHQ-4 is a four-item screen for depression and anxiety that has good sensitivity and specificity for identifying depression and anxiety disorders [22].

##### EQ-5D

The EQ-5D is a standardized health outcome instrument consisting of five dimensions (mobility, self-care,usual activities, pain/discomfort, and anxiety/depression). In addition, the instrument includes a “feeling thermometer” to assess respondents’ current health-related quality of life (0–100). The EQ-5D has been extensively validated and studied for a wide variety of conditions and populations, including the elderly, and is used as a utility measure in cost-effectiveness analyses (http://www.euroquol.com)[23].

##### Behavioral Risk Factor Surveillance System *(BRFSS) Falls*

The BRFSS Falls screen is a two-item questionnaire that assesses the number of falls the respondent has had in the past 3 weeks and how many resulted in injury [24-29].

#### Additional data

**Figure 1:**
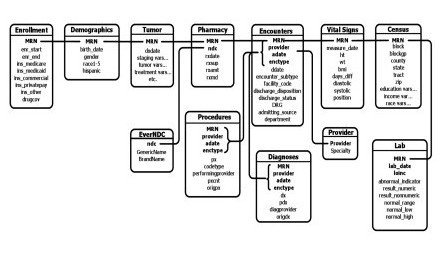
Virtual Data Warehouse data elements.

In addition to the patient-reported outcome measures, we will use electronic medical record and administrative data that are available at these integrated health systems. These health systems have standardized administrative and clinical data collected across their systems (Figure 1). We will generate queries of each health system’s information system to acquire demographic, pharmacy, laboratory, vital sign, and provider data. Table 4 contains a list of variables that we plan provisionally to obtain from each site for each subject.

**Table 4:** Variables to be obtained directly from Health Information Systems

**INDEX VISIT**	**INSURANCE**
Date	Medicare
ICD-9 codes (primary, secondary)	Medicaid
Encounter type	Commercial
Encounter subtype	Private Pay
Facility code	Drug coverage
Provider specialty	Co-pay
Provider age	
Provider gender	
Provider ordering patterns	
**IMAGING**	**LABORATORY DATA**
Date	Date
Ordering physician specialty	Lab test
Indication for image	Result
Image Result (diagnosis)	Normal range
CPT code	
DICOM images de-identified	
**PHARMACY (All medications that we will subsequently categorize relatedness to back pain)**	**PROCEDURES**
Date	Date
Generic name	CPT code
Brand name	Indication for procedure
Supply	Referring physician specialty
Amount	Performing physician specialty
Specialty of treating physician	
Number times refilled	
**PROVIDER VISITS**	**VITAL SIGNS**
Date	Height
ICD-9 codes (primary, secondary)	Weight
Encounter code/type	Blood pressure
Encounter subtype	
Provider specialty	**COMORBIDITY INDEX**
Primary Care	Quan score
Specialty Care	Charlson score
Urgent Care	Elixhauser score
Emergency room	
PT	
Chiropractor	
Massage	
Acupuncture	

#### Data management

For all interviews, research coordinators enter data on specially formatted, paper data collection forms that are stored securely at each site. In addition, sites have the option of entering data directly into the on-line REDCap data system [30]. This has the added advantage of automated range and logic checks that reduce data entry errors. The study has two classes of data: 1) data containing protected health information (PHI) that is only stored locally at each site on a secure server; and 2) a limited data set with dates of service but no other PHI that is uploaded to a central database at the DCC using a web interface.

Research assistants check data from the interviews for missing or unclear responses while the subject is still available. The data coordinating center’s senior programmer directs the data management and re-checks the data for quality. We defined specific logic rules for establishing the internal consistency of responses across several variables. When necessary, we check the original data collection forms or re-contact the subject.

#### Analytic approach

In many registries, there may be a relatively small subgroup of interest (e.g., a treatment or diagnostic testing prevalence of 1 or 2%) and a large number of controls available for a comparative analysis of outcomes. As a general analytic approach in BOLD, we will evaluate cases in comparative studies using 3:1 matched controls with the nearest propensity score [31].

Propensity-based matching is a strategy for assembling similar groups of patients in the absence of randomization. We will use this method to select control patients who are similar to patients who have selectively received the intervention of interest (e.g. early imaging). If we do not appropriately match controls on important baseline characteristics to patients who receive the intervention, there is a risk of obtaining biased results when comparing cases with controls due to confounding [31-37]. Propensity score matching aims to provide a valid estimate of the intervention effect by comparing patients who have and have not had the intervention and who also have similar observed characteristics.

For a given research question and set of patients, we will use logistic regression models (or multi-level logistic regression models for ordinal or multilevel treatments; e.g., levels of treatment dose) to generate a propensity score for each person, using variables that are significant predictors of the intervention of interest. We will then match the propensity score of each case who received the intervention to the nearest propensity scores (up to 3) available among control patients whose propensity scores lie within a caliper window of 0.2σ of the case index, where σ is a measure of variation in the propensity score distributions of cases and controls as given in Rosenbaum and Rubin [35]. If no control propensity scores fall within the caliper window of a particular index case, then the index case will be excluded from any analyses.

### Observational cohort of early imaging

We will conduct an observational cohort study of early imaging in seniors with new visits for back pain as our first comparative study using data from the BOLD registry. Our goal is to test the hypothesis that imaging of the lumbar spine within 6 weeks of the index visit (early imaging) is associated with worse patient outcomes and increased health care utilization and costs. Patients who get early imaging may be those with the worst pain or most alarming clinical presentation. However, given the variability in clinician ordering patterns, there is also a reasonable likelihood that those patients who do and do not get early imaging have considerable overlap.

Prior work has suggested an association between early imaging and subsequent interventions [38,39] but lacked the statistical power to detect a significant association.

#### Subject eligibility

All subjects enrolled in the registry will be eligible for the observational study of early imaging. Cases selected for the observational cohort will be registry patients who had early imaging of the lumbar spine. We will identify propensity score-matched controls from the registry (see below) who did not have early imaging of their spine.

#### Analytic approach to the observational cohort of early imaging

Our overall aim for the observational cohort study is to compare the pain, function, and resource utilization and associated costs of patients who have early (within six weeks of index medical visit) imaging (radiographs, magnetic resonance imaging (MRI), computed tomography (CT) and bone scans) to those who do not have early imaging. The sample will consist of registry patients with new episodes of back pain. Our primary hypothesis is that patients who undergo early imaging will have worse modified RMDQ scores at one year compared with those who do not receive early imaging, after controlling for baseline back pain-related disability, pain severity and pain duration. Our rationale is that imaging may lead to adverse labeling [40] or more interventions (injections, surgery) [39], with resultant complications. We will also test the hypothesis that early-imaged subjects undergo more invasive and more resource-intensive subsequent interventions than those who do not.

#### Matching

We will construct a propensity score based upon the logit function of the probability of receiving early imaging (e.g., the log odds) for a patient with specific characteristics or prognostic factors [37]. We will use fixed matching of age (5-year strata), sex (male/female), and race (Caucasian/African American/other) in the generation of the propensity score and include candidate baseline covariates such as other co-morbidities or diagnoses identified at baseline, modified RMDQ score, and pain intensity rating. Patients receiving early imaging will be matched to the closest control whose propensity score differed by less than 0.2σ among those patients within five years of age.

#### Primary analysis

Our primary outcome measure is back-specific disability measured by the RMDQ at 12 months. We have selected the 12-month assessment as the primary outcome because this allows adequate time for any intervention benefit to manifest, and is the final assessment opportunity for the initial registry study design.

We will first assess comparability of baseline characteristics between the matched groups to gauge the effectiveness of the propensity matching and then address any residual covariate imbalances through model adjustment. Rosenbaum and Rubin suggested that an approach combining both the propensity score and covariate adjustment is superior to the use of either strategy alone [41].

Using the propensity-matched pairs, we plan to use a paired t-test to compare the between-group 12-month change in RMDQ. In conjunction with this primary analysis, we will use multivariate linear regression models adjusting for the propensity score and baseline factors that appear to have residual imbalance in order to compare groups with and without early imaging.

We will use multivariate linear regression models adjusting for the propensity score or conditional logistic models to identify predictors of patient outcome at the one-year follow-up. We will use interaction terms between the early imaging and baseline characteristics to identify variables that predict differences in the outcome associations between the two groups.

We will include subjects who have subsequent imaging more than six weeks after entry to the study in the non-early imaging group. We will compare characteristics of subjects who receive later imaging to those who do not in a sensitivity analysis.

#### Secondary analyses

We will conduct similar analyses for the RMDQ at three and six months as well as for the pain NRS and EQ-5D using all data through one year. We will use methods appropriate for the analysis of repeated measures such as linear mixed models or repeated measures ANCOVA [42], adjusting for the propensity score. We will assess binary secondary outcomes such as achievement of a 30% reduction in pain using conditional logistic regression models.

Using the patient‐reported data and the electronic health system information systems, we will enumerate the number and type of invasive interventions that patients undergo following enrollment. These interventions are listed in Table 5. We will use fixed effects conditional Poisson regression models to compare adjusted spinal surgery rates between those patients who did or did not receive early imaging, conditional on matched pair [43]. In addition, we will examine the time to first invasive intervention using survival analysis with a Cox proportional hazards model and adjust for the propensity score for early imaging.

**Table 5:** Invasive Interventions

Diagnostic	Therapeutic- Percutaneous	Therapeutic- Open Surgical
1. Myelography	1. Epidural steroid injections	1. X-stop
2. Arthrography	2. Facet injection	2. Laminectomy/ discectomy/ decompression
3. Biopsy	3. Trigger point injection	3. Posterior fusion or T-PLIF
		4. Anterior fusion
		5. Anterior and posterior fusion

Another hypothesis is that racial and ethnic minorities will have lower rates of early imaging than non-minorities. To test this hypothesis, we will use the registry to compare rates of early imaging between African Americans/ Blacks and Whites as well as between Hispanics and Whites. We will test for differences in rates using fixed effects conditional Poisson regression models, controlling for the propensity score and residual imbalances among important covariates. We will also examine subsequent invasive interventions as well as outcomes in each of these ethnic and racial subgroups. If early imaging rates are indeed lower in racial and ethnic minorities, we would expect subsequent invasive interventions to be fewer and functional status better.

#### Economic analysis

The primary economic hypothesis is that patients receiving early imaging will have higher health care utilization, higher costs, and worse outcomes at one year compared to those not receiving early imaging. The primary economic outcome will be one-year incremental cost per quality-adjusted life year (QALY) gained from the private/public payer perspective [44].

The cost-effectiveness assessment will use the health systems’ electronic medical records and administrative data as well as patient-reported outcome data. We will use the electronic data to assess within-health system categories of resource utilization (e.g., office visits, procedures, surgeries, tests, medications). We will use the Marketscan® data warehouse (http://marketscan.thomsonreuters.com/marketscanportal/) to obtain an estimate of 2012 private payer average unit costs for medications and medical procedures/services.

We will report short-term costs and consequences (baseline to 3 months) and assess six-month and one-year outcomes incorporating the linear mixed-model approach used in the primary outcomes analysis. Sensitivity and specific scenario analyses will be undertaken to evaluate uncertainty on cost-effectiveness parameters [45].

#### Sample size

Prior studies suggest that approximately 15%–30% of back pain patients will have early imaging of the lumbar spine [46]. Given a registry size of 5,000 subjects, we expect 750–1,500 patients in the BOLD registry will have early imaging and comprise cases for the observational matched cohort study.

In a matched study, missing data at follow-up in either the case or matched control imply that neither patient’s data will be included in a matched analysis. That is, if we anticipate between 10–15% loss to follow-up equally balanced between comparison groups, the number of missing data points can be as much as doubled in any matched or conditional analysis. To compensate for this, we will enrich the control sample with 3:1 matched sampling so that each case will have up to three controls followed in an identical manner. In Table 6, we see that this number of patients offers adequate power to detect minimally clinically relevant differences in functional and pain outcomes, as well as important differences in rates of surgery, complications, or adverse events. Given that one of our enrolling sites (Kaiser Permanente Northern California) is much larger than the other sites, we anticipate approximately triple the number of subjects to be enrolled from KPNC than the other two sites, or 3,000 vs. 1,000 subjects.

**Table 6:** BOLD Power Estimates (registry size = 5,000 patients)*

	Index Case Prevalence			
	2%	3%	5%	10%
**Number of Cases**	100	150	250	500
**Effect Size (D)**				
*RDQ†*				
1.5	-	-	61%	88%
2.0	-	63%	84%	98%
2.5	65%	82%	96%	99%
*Pain NRS†*				
0.7	-	52%	74%	96%
1.0	65%	82%	96%	99%
1.3	87%	96%	99%	99%
*Incremental Cost per QALY Gain < $50,000††*				
$500 / 0.03 **D**QALY	-	-	-	38%
$500 / 0.06 **D**QALY	45%	61%	82%	98%
$800 / 0.03 **D**QALY	-	-	-	21%
$800 / 0.06 **D**QALY	36%	51%	72%	95%
Rare Events (Surgery, Complication, or Binary Event Rate)**				
2% Incidence Rate				
RR = 1.5	-	-	-	-
RR = 2.0	-	-	-	62%
RR = 2.5	-	-	60%	88%
5% Incidence Rate				
RR = 1.5	-	-	-	51%
RR = 2.0	-	51%	73%	95%
RR = 2.5	-	79%	95%	99%

*Comparisons where power estimates are below 50% are not provided

† Conservatively assumes 1:1 case:control ratio, SD estimates of 7.5 and 3.0 for RDQ and Pain NRS respectively.

†† Conservatively assumes 1:1 case to control ratio, calculated for one-year time period, cost standard deviations of $4,000 per group, QALY standard deviations of 0.2 per group based on EQ-5D and 6-month effect improvement, and a positive correlation of 0.3 between costs and changes in EQ-5D [47]. Assuming a negative correlation did not affect power substantially.

**Assumes 3:1 matching of controls to index cases.

An important advantage of a registry is the ability to detect relatively rare events due to the large sample size. We base our sample size estimates on the ability to detect and make inference on relatively rare events. In the primary care setting, examples of rare events would be subsequent surgery or adverse outcomes from interventions such as epidural steroid injections. While our first planned use of the registry is for the comparative effectiveness evaluation of early imaging vs. no-early imaging in the elderly, we envision other evaluations such as the comparative effectiveness of physical therapy vs. no physical therapy.

#### Data access

As the registry progresses in size and maturity, we anticipate making the BOLD resources available to researchers interested in evaluating diagnostic tests, treatments, and outcomes among elderly patients with back pain. Detailed information regarding data sharing will be available at http://www.backpainproject.org.

## Discussion

### Back pain registries

In the U.S., many spine-related registries are device- or procedure-focused and hence recruit patients primarily from specialists. Outside the U.S., several prospective spine registries/cohorts have been established to study various aspects of back pain and while somewhat broader in scope, most still have a specialist focus [48-50]. In contrast, Costa and colleagues established an inception cohort of 973 primary care patients with acute (less than two weeks) low back pain [51], demonstrating both the feasibility and value of such an approach. Identifying patients early in the course allowed measurement of baseline factors that predicted progression and chronicity.

The Back Complaints in the Elders (BACE) consortium is an international group of investigators who have independently established prospective cohorts in a primary care setting to investigate back pain among seniors [52]. Investigators from the Netherlands, Australia and Brazil are collaborating to identify prognostic indicators leading to the transition from acute to chronic back pain in the elderly. The objectives of BOLD parallel those of BACE and similar study structures facilitate international comparisons.

### Strengths of registries

A great advantage of registries is that patient enrollment is easier than intervention trials, so large sample sizes are feasible. This increases generalizability and the ability to detect rare events, such as complications.

#### Limitations of registries

Roovers highlighted the limitations of registries, including lack of proper control groups, confounding, bias, poor data quality control, and potential conflicts of interest due to industry sponsorship [53]. Due to these limitations, registries will never replace randomized controlled trials (RCTs). The most important limitation of registries in general is the lack of a pre-defined control group. However, we can identify important subgroups contained within the registry to use for comparative effectiveness evaluations, such as patients with and without early imaging, and use propensity-matched controls to minimize selection bias associated with treatment or diagnostic testing.

Another limitation of registries is selection bias associated with enrollment. Physicians might be more likely to enroll uncomplicated patients who are likely to have better outcomes. We avoid this shortcoming by using the health information systems to identify potential patients and have a research coordinator contact and enroll them (without prior screening by the primary care physician). Limiting enrollment to integrated health systems somewhat limits generalizability, since the delivery of care within these systems is distinct from the fee-for-service delivery system, with unique incentives. Nevertheless, we believe that the advantages of integrated health systems (comprehensive tracking of healthcare utilization and well-defined population) far outweigh the limitations.

### Diagnostic imaging and back pain in the elderly

Patients and clinicians both tend to under-appreciate the disadvantages of diagnostic testing. The degree of potential controversy associated with this issue was recently emphasized by the release of the U.S. Preventative Health Services Task Force report on mammography [54]. The panel recommended against screening women in the 40–49 year old age group due to the high rate of false positives that could lead to unnecessary further testing and invasive procedures resulting in morbidity without benefit. In addition, these false positives could lead to anxiety and poorer health-related quality of life. Spine imaging in the elderly has similar problems. The rate of incidental findings is high, as high as 90% for some findings [55]. These findings can lead to adverse labeling as well as increased unnecessary interventions, with associated morbidity [40]. Most guidelines exclude patients older than 50 or 65 years from imaging constraints because of the increased prevalence of serious conditions in the elderly. However, it is in the elderly that the rate of incidental lumbar imaging findings is highest [55]. One of our goals is to examine the consequences of early imaging in the elderly by comparing elderly patients who receive early imaging to those who do not.

The BOLD registry establishes an infrastructure for studying back pain in the elderly and performing future comparative effectiveness and cost-effectiveness evaluations. Strengths include accessing patients from a community-based setting and using integrated health plans to facilitate the tracking of resource use. Since the aims and design parallel studies by an international consortium of investigators, comparison with cohorts from the Netherlands, Australia and Brazil will be possible. Even without the potential for future collaborations with international collaborators, the BOLD registry is a valuable new resource for comparative effectiveness research in the United States.

## Competing interests

Dr. Jarvik has the following potential conflicts of interest, although they do not relate directly to the subject of this manuscript, he lists them in the spirit of full disclosure. He serves on the Comparative Effectiveness Advisory Board for GE Healthcare. He is a co-founder and stockholder of PhysioSonics, a high intensity focused ultrasound company, and receives royalties for intellectual property. He is also a consultant for HealthHelp, a radiology benefits management company.

## Authors’ contributions

JGJ, BAC, ALA, BWB, RAD, JLF, PH, LK, SSN, DRN, SDS and JAT developed the original concept of the study and developed the design of BOLD Registry study. ZB and KJ participated in the design of as BOLD and are coordinators. All authors have read and approved the final version of the article.

## Pre-publication history

The pre-publication history for this paper can be accessed here:

http://www.biomedcentral.com/1471-2474/13/64/prepub
